# Impact of postoperative skin disinfection with chlorhexidine on bacterial colonisation following shoulder arthroplasty surgery: a controlled randomised study

**DOI:** 10.1016/j.infpip.2024.100365

**Published:** 2024-04-26

**Authors:** Ida Markström, Karin Falk-Brynhildsen, Margareta Bachrack-Lindström, Gunilla Hollman Frisman, Sara Mernelius, Kristofer Bjerså

**Affiliations:** a Department of Health, Medicine and Caring Sciences, Division of Nursing Sciences and Reproductive Health, Linköping University, Linköping, Sweden; b Department of Anaesthesiology and Intensive Care, Vrinnevi Hospital, Norrköping, Sweden; c Faculty of Medicine and Health, School of Health Sciences, Örebro University, Örebro, Sweden; d Department of Surgery, Clinical Sciences, Sahlgrenska Academy, University of Gothenburg, Gothenburg, Sweden; e Laboratory Medicine, Region Jönköping County, Jönköping and Department of Biomedical and Clinical Sciences, Linköping University, Linköping, Sweden

**Keywords:** Evidence-based practice, Infection control, Patient safety, Prevention, Skin preparation, Surgical site infections

## Abstract

**Background:**

Surgical site infections are a significant threat to patient safety. Shoulder arthroplasty carries an increased risk due to foreign implants. Skin preparation in general is a key preoperative preventive intervention, and the use of chlorhexidine can have a prolonged effect on bacterial colonisation. There is a lack of evidence regarding whether postoperative disinfection has an impact on bacterial colonisation during the first 48 hours after surgery. Our hypothesis was that applying postoperative antiseptic with 5 mg/ml chlorhexidine in 70% ethanol would lead to reduced bacterial colonisation with *Staphylococcus aureus*, coagulase-negative staphylococcus and *Cutibacterium acnes* around the surgical wound within the initial 48 hours after elective shoulder surgery, compared with the use of sodium chloride.

**Methods:**

A single-blinded, controlled study was conducted at a county hospital in Sweden. Swabs from the skin were collected four times: at baseline, preoperatively, after the intervention and after 48 hours.

**Results:**

Our hypothesis was not confirmed. Although not statistically significant, the chlorhexidine group had a higher prevalence of bacterial colonisation of clinically relevant bacteria.

**Conclusions:**

Our study could not confirm that postoperative disinfection with chlorhexidine reduces bacterial colonisation compared with sodium chloride. The results highlight the complexity of SSIs and the importance of evidence-based preventive skin preparation to ensure patient safety. Further research is needed, considering the study's limitations, to explore and evaluate the effectiveness of different skin cleansing solutions and preventive strategies in diverse surgical contexts.

## Introduction

Surgical site infections (SSIs) are infections of the incision, tissue, implanted material, or organs that occur after surgery [1]. SSIs are a major threat to patient safety, cause patient suffering, morbidity, and mortality and represent a substantial economic burden for society [[2], [3], [4]]. Orthopaedic arthroplasty surgery carries an elevated risk due to the potential for SSIs related to the presence of foreign implants. These SSIs can lead to severe problems such as physical disability, and the need for additional surgery [5]. According to national data trends, the incidence rate of shoulder arthroplasty reveals a prevalence of 20 shoulder prostheses per 100,000 inhabitants [6]. The primary indications for shoulder replacement surgery include pain and degenerative conditions of the glenohumeral joint [7]. The reported incidence of SSIs after shoulder arthroplasty ranges from 0.61 to 1.8% [[8], [9], [10], [11]].

The development of an SSI is a complicated process and various factors associated with the patient, the surgical environment, staff behaviour, and surgical technique may impact [12,13]. The cause of SSI is usually skin bacteria, often originating from the patient's own skin flora [14]. *Staphylococcus aureus,* coagulase-negative staphylococci (CoNS) and *Cutibacterium acnes* are the bacteria most involved in SSIs following shoulder arthroplasty [15,16]. *C. acnes* is commonly found in sebum hair follicles and pores on the head, neck, chest, and axilla [17]. The bacterial load, bacterial virulence, and the patient's resistance to infection are critical factors for SSI [14,18].

Nearly half of all SSIs may be prevented through the application of evidence-based strategies [19]. Prevention of SSIs is a complex process that necessitates the integration of a variety of preventive actions before, during, and after surgery [13,20]. Skin preparation aimed at decreasing bacterial colonisation at the surgical site is one of the most important preoperative preventive interventions [[21], [22], [23]]. Worldwide, different antiseptics are used in skin preparation, and ethanol combined with chlorhexidine is recommended by several SSI preventive guidelines [21,22]. The combination of ethanol and chlorhexidine effectively eliminates most bacteria, fungi, and viruses, and when applied repeatedly, it can have a lasting impact on bacterial colonisation for multiple days [24,25]. Chlorhexidine covalently binds to proteins in the skin and mucosa, resulting in a prolonged antimicrobial effect [26].

A dressing is necessary during the first 48 hours before the body naturally establishes a protective barrier against environmental factors [27,28]. To ensure proper adhesion of the dressing, cleaning and drying the skin before applying it is essential. Antiseptic agents are sometimes used. There is a lack of scientific evidence for postoperative skin cleansing solutions and their impact on postoperative bacterial colonisation. Currently, different solutions such as sodium chloride or ethanol with or without chlorhexidine are used to clean around the sutured wound, often based on the healthcare provider's personal opinion or according to local guidelines. Considering chlorhexidine's prolonged effect, the application of chlorhexidine around the surgical wound could potentially lower the risk of bacterial presence during the important initial 48 hours. To our knowledge, this has not been investigated previously. The hypothesis was that postoperative disinfection around the surgical wound using 5 mg/ml chlorhexidine in 70% ethanol would result in reduced bacterial colonisation within the initial 48 hours after elective shoulder surgery when compared with the use of sodium chloride. This study has the potential to make a contribution to the development of evidence-based preventive interventions aimed at enhancing patient safety.

## Methods

### Study design

This was a single-blinded, controlled, block-randomised intervention study with random allocation to a chlorhexidine group or a sodium chloride group.

### Sample and setting

Participants were recruited at one county hospital in the southeast of Sweden from August 2019 to March 2023. Consecutive sampling was used, and adult patients over 18 years of age scheduled for primary elective shoulder arthroplasty surgery were approached for participation. Exclusion criteria were a previous allergic reaction or perceived adverse effects of chlorhexidine, prolonged treatment with cortisone (>5 mg) for more than five days for the last month, antibiotic treatment within 14 days prior to scheduled surgery, skin disease or ongoing skin infection. The participants had to be able to understand Swedish in both spoken and written form. The trial was retrospectively registered at ClinicalTrials.gov (Identifier: NCT06114459).

### Data collection

#### Recruitment and randomisation

**Figure 1:**
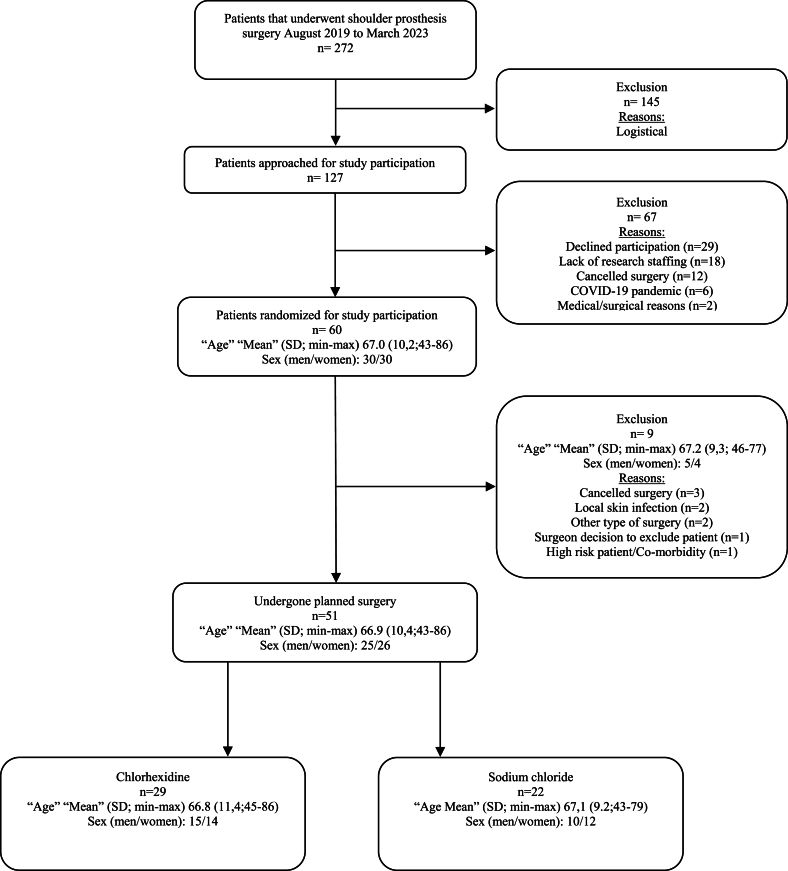
Flow chart of study subject, exclusion and dropouts.

With the help of an administrator, an invitation for participation was sent to all eligible patients along with the surgical appointment. The first author made a phone call to potential participants to provide additional information and to obtain oral consent. An external statistician produced the randomisation using computer-generated block randomisation, with participants assigned in blocks of ten. Patients were randomly assigned to chlorhexidine or sodium chloride groups (Figure 1). Sealed envelopes were used for the randomisation process. Patients and staff at the laboratory were blinded throughout the entire study, while the operating room (OR) nurse was blinded until the intervention.

#### Procedure

All orthopaedic OR nurses at the department were educated and clinically trained regarding the study and the intervention. Trained OR nurses, assisted by either another OR nurse or a nurse assistant, conducted all sample collections and interventions. A case report form (CRF) was created containing background data and signature boxes for sampling and follow-up. The study design and CRF were pretested, and some minor changes were made to the CRF and the intervention. These results are not included.

Patients in the study followed a standard care programme, including two preoperative showers using a 40 mg/ml chlorhexidine gluconate soap solution, Descutan® (Fresenius Kabi AB, Uppsala, Sweden); one shower the night before and one on the morning of surgery. Upon arrival at the preoperative department, an OR nurse obtained written informed consent, collected demographic information, and conducted the baseline sampling (see Samples). All patients received a shoulder nerve blockade, preceded by skin antisepsis using 5 mg/ml chlorhexidine in 70% ethanol by the anaesthesiologist. Thereafter, a local wash of the shoulder was performed using a soap solution containing 40 mg/ml chlorhexidine gluconate, Hibiscrub® (Mölnlycke, Sweden). Immediately before incision, the OR nurse disinfected the surgical site with 5 mg/ml chlorhexidine in 70% ethanol and draped it with sterile materials, including an iodophor-impregnated incision drape (Ioban2, 3M, Sweden). After draping, the second sample was collected. Once the surgery was completed and the skin closure had been performed, the OR nurse cleaned the surgical site with sodium chloride. Next, the sealed envelope was opened, and the intervention was performed (see Intervention). Following the intervention, the third sample was collected, and a wound dressing (Mepilex Border Postop®, Mölnlycke, Sweden) was applied. Approximately 48 hours post-surgery, the fourth sampling was conducted under sterile conditions. The OR nurse assessed the surgical wound for infection signs, and patients reported pain using the Visual Analog Scale and any dressing changes.

#### Intervention

**Figure 2:**
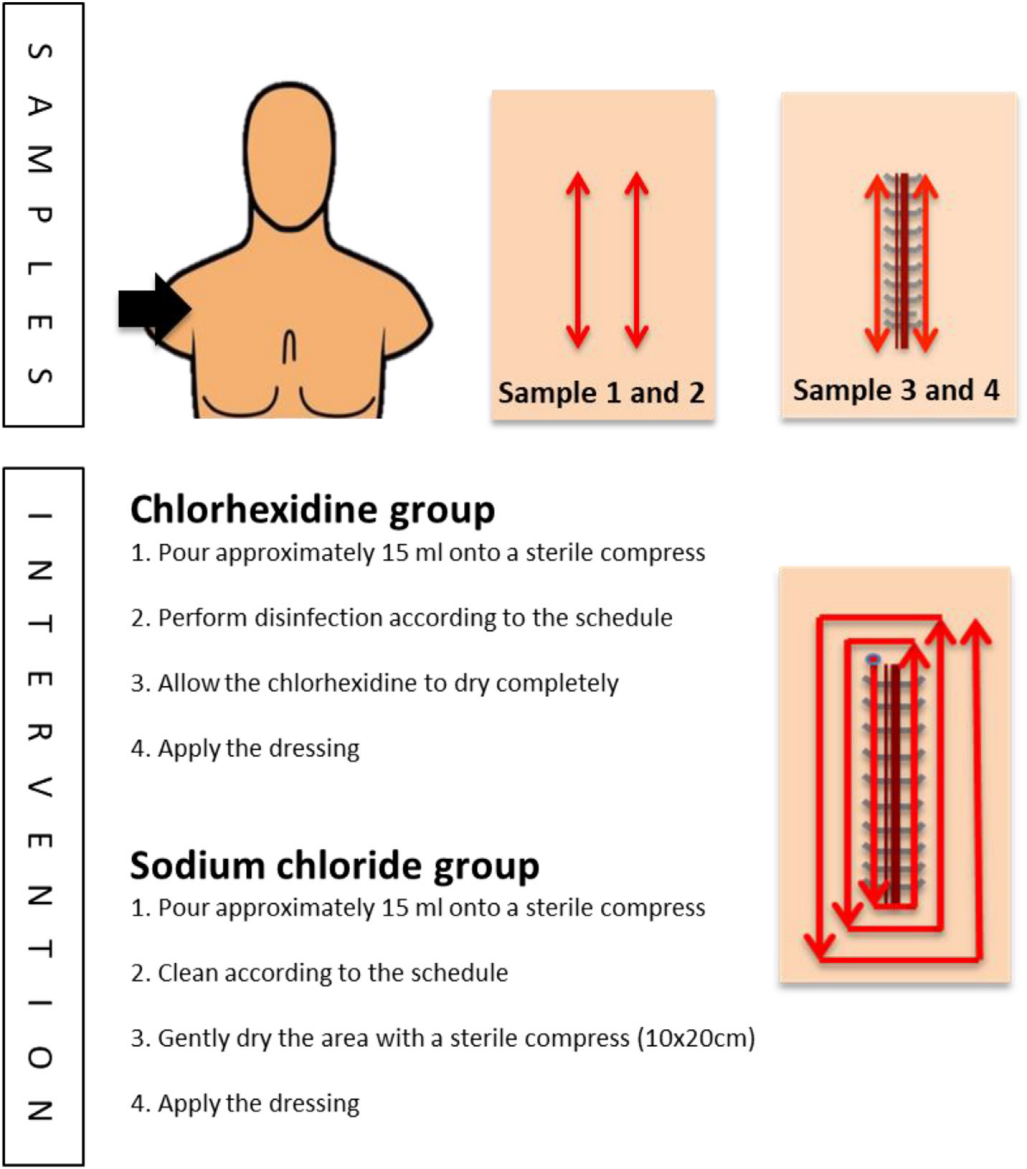
Site for samples and instruction for intervention.

According to a predetermined schedule, the surgery site was either disinfected with 5 mg chlorhexidine in 70% ethanol or cleaned with sterile sodium chloride (Figure 2).

#### Samples

Swab-samples from the skin were collected on four occasions: 1. At baseline, 2. After disinfection, 3. Post-intervention, and 4. After 48 hours. All samples collected in the study were collected utilising the eSwab system with flocked swabs (Copan Italia S.p.A. via Perotti 10, Brescia, Italy) (Figure 2). On each occasion, two samples were collected using the Pencil Eraser Swab technique [29]. Swabs were gently rubbed in an oscillating motion, moving downward, and then replicating the same motion upward, repeated 15 times. For samples 1 and 2, one swab on each side of the intended incision site was collected. For samples 3 and 4, samples were taken from each side of the incision, positioned 1 cm away from the sutures or staples, with one swab used for each side (Figure 2).

The laboratory personnel followed standard procedures in accordance with Good Laboratory Practice for handling collected samples. Samples were cultivated on two blood agar plates (21 ml each in 9 cm petri dishes, 1,000 g Difco Columbia Blood Agar Base (279240), (Becton, Dickinson and Company, Franklin Lakes, NJ), 22,700 ml deionised water, and 1,500 ml ± 150 ml human blood) and one hematin agar plate (440 g Difco Columbia Blood Agar Base (Becton Dickinson and Company, Franklin Lakes, NJ), 10,000 ml deionised water, 820 ml defibrinated horse blood (allowed to haemolyse) and 1,000 ml horse serum). One blood agar plate was incubated aerobically and the haematin agar plate was incubated aerobically with the addition of 5 % CO_2_, both at 36 °C. Both plates were examined after one and two days. The other blood agar plate was incubated anaerobically at 36 °C for ten days. Species determination was performed using MALDI-ToF technology (MALDI Biotyper® Sirius System, Bruker, Billerica, MA).

#### Measurements

Postoperative bacterial colonisation was dichotomously assessed as either “growth” or “no growth”. The first author reviewed the patients' medical records and followed up with participants by phone for a duration ranging from six to twenty-five months, with a median follow-up period of 21 months after the procedure in order to look for potential complications and SSIs. Variability in follow-up timelines was influenced by the COVID-19 pandemic.

#### Sample size calculations

The sample size calculation was based on an earlier study [30]. Based on their findings, the sample size calculation aimed to detect a 10% difference in positive bacterial colonisation between patients in the chlorhexidine group and the sodium chloride group within 48 hours post-surgery. It was estimated that a sample of 60 patients would have a power of 80% at a significance level of 5% to detect these differences. Fisher's exact test, one-tailed, was performed using G∗Power 3.1 Software. To account for potential internal dropouts and ensure an adequate number of participants, a decision was made to include 100 patients.

#### Statistical analysis

The data was compiled using Microsoft Excel® and analysed utilising IBM Statistical Package for the Social Sciences version 29 (New York, NY). Descriptive statistics were utilised to discern disparities among the groups in terms of background characteristics, involving numerical values, means and standard deviations. Frequencies were described in numbers and percentages. Differences between groups (age, body mass index, time in surgery, time for antibiotic prophylaxis, and surgery type) were compared using an Independent student t-test. For comparison of proportions for categorical data such as sex, nicotine use, morbidity, preparations, and skin closure, the Pearson Chi-square test or Fisher's exact test were used. Postoperative bacterial colonisation levels were compared using the Pearson's Chi-square test or Fisher's exact test. A statistical level of *P*< 0.05 was considered significant.

#### Amendments

During the study period, the prevailing conditions of COVID-19 required temporary reassignment of healthcare personnel to other departments for the care of COVID-19 patients. Consequently, in March 2020, the surgical schedule at the study site experienced significant disruptions, resulting in a reduction in planned surgical procedures. When the study resumed in September 2021, the surgical pace was noticeably diminished due to logistical reasons, significantly affecting the rate of participant inclusion. In March 2023, the decision was made to end the study with 60 patients, without accounting for originally planned dropouts.

## Results

Out of the 60 participants included, 51 could be included in the results. Internal dropouts occurred due to various reasons, including emergency surgery, alternative surgical procedures, skin injuries, morbidity, and staff shortages. Out of the nine dropouts, eight were from the sodium chloride group. There were no significant differences in background data between the sodium chloride dropout group and the sodium chloride group. Demographic and background data are presented in Table I.

**Table I:** Demographics, procedures and follow-up

			Chlorhexidine (n=29)	Sodium chloride (n=22)	*P*-value (CI)
**Preop**	Sex, in %, (n=)	Men	51.7 (15)	45.5 (10)	0.657^a^
		Women	48.2 (14)	54.5 (12)	
	Age in years, Mean [SD]		66.8 [11.4]	67,1 [9.2]	0.908^b^
	Prosthesis type Anatomic/Reverse, in % (n=)	Anatomic	41.4 (14)	40.9 (9)	1.000^a^
		Reverse	58.6 (25)	59.1 (13)	
	Body Mass Index, Mean [SD]		30.0 [4.2]	28.0 [3.5]	0.103^b^
	Smoking in %		0	0	-
	Nicotine snuff, daily, in %, (n=)		13.8 (4)	18.2 (4)	0.713^c^
	Diabetes mellitus type II in %, (n=)		10.3 (3)	13.6 (3)	0.524^c^
**Periop**	Shower with chlorhexidine gluconate, in %		100	100	-
	Duration of surgery in minutes, Mean [SD]		96 [24.1]	98 [18.6]	0.576^b^ (-8.955-15.933)^d^
	Time of prophylactic antibiotics before incision in minutes, Mean [SD]; min-max		34.5 [15]; 15-78	33.7 [9]; 11-52	0.829^b^ (-7.553-6.428)^d^
	Hair removal with hairclipper, in %, (n=)		27.5 (9)	40.1 (8)	0.377^a^
	Skin closure, in %, (n=)	Staplers	96.6 (28)	95.4 (21)	
		Intracutaneous suture	0	4.5 (1)	0.354^a^
		Cutaneous suture	3.5 (1)	0	
**Postop**	Early dressing change <48 h, in %, (n=)		7.7 (2)	16.7 (1)	1.000^c^
	Deep wound infection, in %, (n=)		3.5 (1)	0	1.000^c^
	Superficial wound infection, in %, (n=)		3.5 (1)	8.3 (1)	1.000^c^

a Pearson's Chi-square, 2-sided.

b Independent student t-test, paired.

c Fisher's exact test, 2-sided.

d CI= confidence interval (lower-upper).

No statistically significant differences in background data and demographics were observed between the chlorhexidine group and the sodium chloride group. Antibiotic prophylaxis was administered promptly in accordance with the clinic's policy, within 30–45 minutes before incision, except in two cases in the chlorhexidine group and one in the sodium chloride group.

### Bacterial cultures

Bacterial colonisation with the most clinically relevant bacteria for SSIs in prosthesis surgery (*S. aureus,* CoNs, and *C. acnes*) is presented in Table II. The bacterial colonisation of all bacteria is provided in Supplementary file A1. CoNS detected in this study were *Staphylococcus capitis, Staphylococcus caprae, Staphylococcus epidermidis, Staphylococcus hominis, Staphylococcus saccharolyticus and Staphylococcus schleiferi.*

**Table II:** Positive bacterial colonisation

	Type of bacteria in positive lateral, medial or both samples	Percent of patients with positive bacterial cultures %, (n=)		*P*-value
		Chlorhexidine	Sodium chloride	
Preoperative	Any type of bacteria	86.2% (n=25/29)	81.8% (n=18/22)	0.713^b^
	At least one of the three clinically relevant bacteria^a^	79.3% (n=23/29)	77.3% (n=17/22)	1.000^b^
	Coagulase negative staphylococci	55.2% (n=16/29)	59.1% (n=13/22)	0.780^c^
	*Cutibacterium acnes*	65.5% (n=19/29)	72.7% (n=16/22)	0.302^c^
	*Staphylococcus aureus*	3.5% (n=1/29)	0% (n=0/22)	-
After skin disinfection	Any type of bacteria	34.5% (n=10/29)	9.1% (2/22)	0.048^b^
	At least one of the three clinically relevant bacteria^a^	31.0% (n=9/29)	9.1% (n=2/22)	0.088^b^
	Coagulase negative staphylococci	6.9% (n=2/29)	4.5% (n=1/22)	1.000^b^
	*Cutibacterium acnes*	31.0% (n=9/29)	9.1% (n=2/22)	0.059^b^
After intervention	Any type of bacteria	51.7% (n=15/29)	50% (n=11/22)	0.903^c^
	At least one of the three clinically relevant bacteria^a^	51.7% (n=15/29)	54.5% (n=12/22)	0.842^c^
	Coagulase negative staphylococci	6.9% (n=2/29)	27.7% (n=6/22)	0.063^b^
	*Cutibacterium acnes*	44.8% (n=13/29)	45.5% (n=10/22)	1.000^b^
Postoperative >48 hours	Any type of bacteria	50.0% (n=13/26)	41.7% (n=5/12)	0.632^c^
	At least one of the three clinically relevant bacteria^a^	46.2% (n=12/26)	41.7 (n=5/12)	0.796^c^
	Coagulase negative staphylococci	23.1% (n=6/26)	16.7% (n=2/12)	1.000^b^
	*Cutibacterium acnes*	42.3% (n=11/26)	33.3% (n=4/12)	0.728^b^

a Coagulase negative staphylococci, *C. acnes, S. aureus*.

b Fisher's exact test, 2-sided.

c Pearson's Chi-square, 2-sided.

The hypothesis that postoperative antisepsis of the surgical wound with 5 mg/ml chlorhexidine in 70% ethanol would reduce positive bacterial colonisation compared to cleaning with sodium chloride during the initial 48 hours following elective shoulder surgery could not be confirmed. Although the difference was not statistically significant, the chlorhexidine group had a higher prevalence of positive bacterial cultures.

The detailed results are provided in Table II. In the baseline samples, bacterial colonisation was detected in 25 of 29 patients (86%) in the chlorhexidine group and 18 of 22 patients (82%) in the sodium chloride group. Among these, 79% (n=23/25) and 77% (n=17/18) were clinically relevant. In one baseline sample, a single colony of *S. aureus* was detected. Subsequently, no further colonisation with *S*. *aureus* was recognised in any samples. After antiseptics, fewer patients in the sodium chloride group had positive cultures compared to patients in the chlorhexidine group (*P*=0.048). The chlorhexidine group had fewer positive samples of CoNs immediately after the intervention, but this difference was not significant. In both groups, *C. acnes* was found in all four sampling phases.

### Clinical outcomes

Three of the 51 randomised patients were assessed with SSI according to the medical charts, one deep infection and two superficial infections. In the chlorhexidine group, one patient experienced a deep infection and underwent surgery with debridement, antibiotics, and implant retention (DAIR) and cultures showed the growth of *S. aureus*. This patient had one observed colony of *C. acnes* in one baseline sample and no further positive samples within this study. A superficial cutaneous infection was suspected for one patient in the chlorhexidine group; this was treated with antibiotics and no cultures were taken. Due to discolouration around the surgical wound, one patient in the control group was followed up with infection analyses, but no further action was taken.

## Discussion

The expected decrease in positive bacterial colonisation within the chlorhexidine group after 48 hours was not evident. On the contrary, there were indicative findings of lower bacterial colonisation levels in the sodium chloride group. Without significant results, the result indicating that the chlorhexidine group may have had a higher prevalence of positive bacterial samples of clinically relevant bacteria compared to the sodium chloride group. The chlorhexidine group had fewer positive samples of CoNs immediately after the intervention, despite the absence of significant results.

Treatment with chlorhexidine around the wound postoperatively does not seem to have an impact on the postoperative colonisation of clinically relevant bacteria. However, it does appear that using chlorhexidine perioperatively is effective against *S. aureus*, regardless of postoperative interventions like the intervention in the study at hand. The prolonged effect of preoperative skin preparation after treatment with chlorhexidine from a preoperative shower, local cleansing and antisepsis appears to be sufficiently effective, as colonies of *S. aureus* were not found on the skin in any of the groups. This is similar to other studies where bacterial colonisation has been measured following skin preparation [30,31].

Similar studies of postoperative disinfection or cleaning have not been identified. However, several previous studies have investigated perioperative colonisation of the clinically relevant bacteria, with results supporting our study's findings. After 120 minutes of cardiac surgery, *C. acnes* was found on the skin in 44.4% of the patients, while CoNS were present in 23.8% [30]. Similar occurrences of bacterial colonisation were observed before wound closure during pacemaker insertion, with an average surgical duration of 34 minutes, with CoNS at 25.9% and *C. acnes* at 46.4% [31].

Postoperative disinfection with chlorhexidine does not appear to decrease the postoperative colonisation of CoNS; instead, there is an indication that it may increase after 48 hours. Is it possible that chlorhexidine disrupts the skin's protective balance and provides space for other microorganisms? This needs to be further investigated. In this study, chlorhexidine disinfection appears to remove CoNS temporarily.

An important factor to consider is the susceptibility of bacteria to chlorhexidine. Resistance to chlorhexidine in staphylococci strains has been detected in bacteria isolated from prosthetic joint infections [32,33]. Excessive usage of chlorhexidine may promote resistance development, so it is imperative to use chlorhexidine in adherence to evidence-based guidelines.

This study suggests a limited effect of chlorhexidine on *C. acnes,* since colonisation was seen in all stages of sampling, in both groups. This is consistent with previous studies, which have highlighted that conventional surgical skin preparation does not eradicate *C. acnes* from the skin or from sebaceous glands in subdermal layers [[34], [35], [36], [37], [38]]. In one study, *C. acnes* was identified in samples from subdermal layers, the surgeon's glove tips, and scalpel blades [37]. This implies that these bacteria could potentially originate from the edges of surgical incisions and could be disseminated through the surgical technique employed, highlighting the importance of addressing bacteria not only on the skin but also within the skin. Treatment with benzoyl peroxide has been proven effective in reducing *C. acnes* [34,39]. Preoperative topical application of benzoyl peroxide to surgical areas could be considered before surgery [40]. To conduct more studies using benzoyl peroxide instead of chlorhexidine or in combination could be of interest.

Although clinically relevant bacteria were present, our study and similar studies demonstrated a low incidence of SSIs. Not all bacteria are pathogens and this underscores the importance of working in care bundles to address all risk factors, including the patient's ability to resist SSI and the environment surrounding the patient [13,41].

The study's findings indicate that preventive interventions performed in connection to surgery lack evidence-based support and do not yield the desired effect. Despite this study, the clinical issue of using different solutions without a scientific basis continues to persist. Various solutions for postoperative cleaning and antisepsis are employed based on individual preferences, traditions, or local guidelines. These interventions are not based on evidence, which may jeopardise patient safety. Postoperative cleaning or disinfection is an example of a clinical interventions carried out with good intentions. However, it may not have any effect or, in the worst case, could worsen the situation.

This study has limitations. The small sample size was a notable issue, and like other clinical studies conducted between 2020 and 2022, progress was significantly affected by the impact of COVID-19, leading to delays in scheduling and even surgery shutdowns [42]. No external research personnel were allocated, which contributed to the high exclusion rate (n=145) from the initial total sample (Figure 1). Unplanned renovation of the OR clinic was another contributing factor.

Deviation from the initial study plan occurred as patients were discharged from the hospital within less than 48 hours after surgery, resulting in the exclusion of the final evaluative sample. In the sodium chloride group, a comprehensive evaluation with all samples was administered to only 12 out of 22 patients. Due to various reasons described in the results, only 51 patients underwent the intervention. The decision to conclude the study after enrolling 60 patients instead of 100 resulted in an underpowered study, due to significant internal dropouts in the sodium chloride group. Consequently, there is a risk of Type 2 errors, and there might have been differences that we failed to demonstrate. Hence, the results must be interpreted with caution. Finally, this study was conducted in a single centre with a standardised care programme that included practices, logistics, and analysis. Therefore, the results may not be generalisable or applicable to other settings.

Further studies are necessary to assess the effect of postoperative solutions on the skin. Future research could involve a well-powered multicentre study aimed at assessing the efficacy of integrating preoperative treatment with benzoyl peroxide, skin antisepsis using chlorhexidine, and postoperative cleaning employing sodium chloride. Alternatively, a study could be conducted on patients undergoing surgery in different anatomical regions where the prevalence of *C. acnes* is not as common.

## Conclusions

The current study did not confirm the hypothesis that postoperative disinfection with chlorhexidine compared with sodium chloride reduces bacterial colonisation after shoulder arthroplasty surgery. Interestingly, there was an indication suggesting an unexpected increase in positive clinically relevant bacterial samples with the use of chlorhexidine.This unresolved clinical question highlights the importance of offering safe, evidence-based interventions to surgical patients. To ensure patient safety and to obtain a comprehensive assessment of intervention effectiveness, further research using larger, well-designed, and adequately powered controlled trials is needed.

## Ethics approval

The study was approved by the Regional Ethical Review in Linköping Dno 2018/433-31, Dno 2021-06686-02.

## Conflict of interest statement

None.

## Funding statement

This study was founded by Medical Research Council of Southeast Sweden and the County Council of Region Östergötland.

## Author contributions

All authors planned the design of the study. IM led the project. All authors were involved in manuscript drafting and editing. All authors made the final approval of the version to be published and agreed to be accountable for all aspects of the work in ensuring that questions related to the accuracy or integrity of any part of the work are appropriately investigated and resolved.

## Appendix A Supplementary data

Multimedia component 1 All findings of bacteria Multimedia component 1
