# Supplementary material for: Impact of postoperative skin disinfection with chlorhexidine on bacterial colonisation following shoulder arthroplasty surgery: a controlled randomised study
Source: Infect Prev Pract. 2024 Apr 26;6(2):100365. doi: 10.1016/j.infpip.2024.100365 (PMC11098957; doi:10.1016/j.infpip.2024.100365)
Supplement: Multimedia component 1 — All findings of bacteria [file mmc1.docx]

| Time period |  | Chlorhexidine* | Sodium chloride* |
| --- | --- | --- | --- |
| Preoperative | **Positive bacteria samples (Lateral, Medial or both)** | **86.2%, n_Total_=29** | **81.8% n_Total_=22** |
|  | *Actinomyces* | n=1  (single=1) |  |
|  | *Streptococcus mitis* | n=1  (single=1) | n=1  (single=1) |
|  | *Bacillus cereus* | n=1  (single=1) | n=1  (single=1) |
|  | *Brevundimonas* | n=1  (single=1) |  |
|  | *Corynebacterium accolens* | n=1  (single=1) |  |
|  | *Corynebacterium lipophiloflavum* | n=1  (single=1) |  |
|  | *Corynebacterium minutissimum* |  | n=1  (single=1) |
|  | *Coryne bacterium propinquum* | n=1  (single=1) |  |
|  | *Corynebacterium tuberculostearicum* |  | n=1  (single=1) |
|  | *Cutibacterium acnes* | n=19  (single=8; few=5; moderate=5; plenty=1 | n=15  (single=5; few=;6 moderate=2; plenty=2) |
|  | *Deinococcus* | n=1  (single=1) |  |
|  | *Dermabacter* |  | n=1  (single=1) |
|  | *Enterococcus faecalis* |  | n=1  (single=1) |
|  | *Fictibacillus* | n=1  (single=1) |  |
|  | *Fusobacterium nucleatum* | n=1  (moderate=3) |  |
|  | Gram-positive cocci | n=1  (moderate=3) |  |
|  | *Moraxella* | n=2  (single=2) |  |
|  | *Pseudomonas monteilii* |  | n=1  (single=1) |
|  | *Staphylococcus aureus* | n=1  (single=1) |  |
|  | *Staphylococcus capitis* | n=12  (single=10; few=2) | n=4  (single=3; few= 1) |
|  | *Staphylococcus epidermidis* | n=11  (single=7; few=3; moderate=1) | n=8  (single=5; few=3) |
|  | *Staphylococcus hominis* | n=2  (single=1; few=1) | n=5  (single=3; few=2) |
|  | *Staphylococcus saccharolyticus* | n=2  (single=1; few=1) | n=2  (single=1; moderate=1) |
|  | *Streptococcus salivarius* | n=1  (single=1) |  |
|  | *Staphylococcus schleiferi* | n=1  (single=1) |  |
|  | *Streptococcus sanguinis* | n=1  (single=1) |  |
|  | *Micrococcus luteus* | n=10  (single=8; few=2) | n=7  (single=4; few=2; moderate=1) |
|  | *Rothia* |  | n=1  (single=1) |
| Perioperative  before intervention | **Positive bacteria samples (Lateral, Medial or both)** | **34,5%, n_Total_ =29** | **9,1%, n_Total_ =22** |
|  | *Bacillus* | n=1  (single=1) |  |
|  | *Corynebacterium accolens* | n=1  (single=1) |  |
|  | *Corynebacterium propinquum* | n=1  (moderate=1) |  |
|  | *Cutibacterium acnes* | n=10  (single=6; few=2; moderate=2) | n=2  (single=1; few=1) |
|  | *Paenibacillus* | n=1  (single=1) |  |
|  | *Staphylococcus capitis* | n=2  (single=2) | n=1  (single=1) |
|  | *Staphylococcus epidermidis* | n=2  (single=2) |  |
|  | *Staphylococcus sacchaolyticus* | n=1  (few=1) |  |
|  | *Micrococcus luteus* | n=2  (single=2) | n=1  (single=1) |
|  | **Positive bacteria samples (Lateral, Medial or both)** | **44,8%, n_Total_ =29** | **50,0 %, n_Total_ =22** |
| Perioperative  after  intervention | *Cutibacterium acnes* | n=14  (single=5; few=3; moderate=6) | n=10  (single=2; few=3; moderate=4; plenty=1 |
|  | *Cutibacterium granulosum* | n=1  (moderate=1) | n=1  (single=1) |
|  | *Staphylococcus capitis* |  | n=1  (single=1) |
|  | *Staphylococcus caprae* | n=1  (single=1) |  |
|  | *Staphylococcus epidermidis* |  | n=3  (few=2; plenty=1) |
|  | Staphylococcus saccharolyticus | n=1  (few=1) | n=3  (single=1; few=2) |
| Postoperative 48h | **Positive bacteria samples (Lateral, Median or both)** | **50%, n_Total_ =26** | **41,7%, n_Total_ =12** |
|  | *Aerococcus* | n=1  (single=1) |  |
|  | *Anaerobic bacteria* | n=1  (single=1) |  |
|  | *Cutibacterium acnes* | n=11  (single=4 few=2; moderate=4; plenty=1) | n=4  (single=2; few=2) |
|  | *Kleibsiella aerogenes* | n=1  (moderate=1) |  |
|  | *Staphylococcus capitis* | n=2  (single=1; few=1) |  |
|  | *Staphylococcus epidermidis* | n=2  (single=2) | n=1  (moderate=1) |
|  | *Staphylococcus saccharolyticus* | n=1  (moderate=1) | n=1  (few=1) |
|  | *Staphylococcus schleiferi* | n=1  (single=1) |  |
|  | *Micrococcus luteus* |  | n=1  (single=1) |

*Value in brackets, Single = 1-5 colonies; Few = 6-50 colonies, Moderate= >50 colonies, Plenty= unable to distinguish individual colonies
